# Single-nucleus rna sequencing identifies universal camk1d upregulation and dysregulated c-ltmr subtypes as key drivers of paclitaxel-induced neuropathy

**DOI:** 10.1007/s10565-025-10065-z

**Published:** 2025-07-07

**Authors:** Wuping Sun, Rongzhen Li, Xinyi Zhang, Songbin Wu, Yanjun Jiang, Qian Li, Di Cao, Donglin Xiong, Lizu Xiao, Xiaodong Liu

**Affiliations:** 1 Department of Pain Medicine and Shenzhen Municipal Key Laboratory for Pain Medicine, Shenzhen Nanshan People’s Hospital and the 6Th Affiliated Hospital of Shenzhen University Medical School, Shenzhen, 518060 China; 2 Department of Anaesthesia and Intensive Care, The Chinese University of Hong Kong, Hong Kong SAR, China; 3 Peter Hung Pain Research Institute, The Chinese University of Hong Kong, Hong Kong SAR, China

**Keywords:** Chemotherapy-induced peripheral neuropathy, Paclitaxel-induced peripheral neuropathy, Mechanical allodynia, Thermal hyperalgesia, Cold hyperalgesia, Single nuclei RNA sequencing

## Abstract

Neuropathic pain triggered by chemotherapy poses a significant clinical challenge. Investigating cell type-specific alterations through single-cell transcriptome analysis holds promise in understanding symptom development and pathogenesis. In this study, we performed single nuclei RNA (snRNA) sequencing of dorsal root ganglions (DRG) to explore the molecular mechanism underlying paclitaxel-induced neuropathic pain. Mouse exposed to repeated paclitaxel doses developed persistent pain hypersensitivity lasting at least 21 days. The snRNA sequencing unveiled seven major cell types within DRGs, with neurons further subdivided into 12 distinct subclusters using known markers. Notably, type C low-threshold mechanoreceptors (C_LTMR) exhibited the most pronounced transcriptomic changes post-paclitaxel administration. Differential gene expression and Gene Ontology (GO) analysis highlighted suppressed potassium-related currents, microtubule transport, and mitochondrial functions in C_LTMR following paclitaxel treatment. Pseudo-time analysis uncovered nine distinct states (state 1 to 9) of C_LTMR. State 1 exhibits higher prevalence in paclitaxel-treated mice and altered neurotransmission properties, likely contributing to paclitaxel-induced pain hypersensitivity. Additionally, Camk1d is involved in temperature hyperalgesia in CIPN, a key clinical symptom observed in human patients with CIPN. This comprehensive exploration sheds light on the molecular mechanisms driving paclitaxel-induced neuropathic pain, offering potential avenues for therapeutic intervention.

## Introduction

Peripheral neuropathy commonly emerges as an unfavorable consequence of anticancer therapies utilizing various chemotherapeutic agents such as paclitaxel, oxaliplatin, and vincristine. This type of neuropathy is typically manifested by neuropathic symptoms such as numbness and abnormal sensations in the extremities, particularly increased pain perception to mechanical and temperature stimuli, and is therefore referred to as chemotherapy-induced peripheral neuropathy (CIPN) (Boyette-Davis et al. 2011; Zhi et al. 2019). CIPN impacts approximately 30–40% of patients receiving cancer treatments, often leading to an unintended decrease in therapeutic dosage or even discontinuation of treatment (Sisignano et al. 2014; Staff et al. 2017). In some cases, CIPN can endure for months to years after treatment completion, severely impacting patients'quality of life (Sisignano et al. 2014). Effective prevention and treatment of CIPN stands as a medical necessity.

Currently, there are no effective clinical strategies to counteract CIPN (Hershman et al. 2014; Loprinzi et al. 2020). The obstacles in developing CIPN-related drugs have several reasons. First, the mechanisms of CIPN are intricate and not entirely understood. Chemotherapy treatments induce multifaceted structural and functional changes in non-cancerous cells, resulting in damage to cell organelles (e.g. cytoskeleton and mitochondrion), altered signaling pathways, changes in membrane receptors and ion channels, and modulation of neurotransmitter levels. These alterations impact the RNA and protein profile of both primary sensory neurons and glial cells and influence the excitability and firing threshold of primary sensory neurons (Areti et al. 2014; Canta et al. 2015; Khuankaew et al. 2021; Sahu et al. 2021; Trecarichi and Flatters 2019; Zajaczkowska et al. 2019), contributing to the development of CIPN. In our prior study, we conducted transcriptome analysis of dorsal root ganglions (DRG) in oxaliplatin- and paclitaxel-induced CIPN. We revealed substantial distinctions in the pathogenesis of these two CIPN models. Specifically, paclitaxel tended to directly impact somatosensory neurons, while oxaliplatin exhibited a propensity to affect glial cells (Sun et al. 2023a, 2023b). Second, the presence of cancers, which might share common or similar signaling pathways but have opposing effects on CIPN, adds to the complexity of the pathogenesis of CIPN (Zajaczkowska et al. 2019). Preventing CIPN without compromising chemotherapy efficacy presents significant operational challenges. Understanding CIPN-specific mechanisms is pivotal for developing clinical management strategies, holding significant scientific and clinical translational value.

Recent advancements in omics at a single-cell resolution not only elucidate the cellular atlas of pain pathways but also unveil cell-type-specific alterations. These insights enhance our comprehension of symptom emergence, such as tactile allodynia and thermal pain, and reveal dysregulated genes that might have been overlooked in bulk tissue-based analyses (Li et al. 2016; Wang et al. 2021b). These discoveries undoubtedly introduce a novel dimension to our understanding of the pathogenesis of CIPN and the identification of targets/signaling pathways specific to subtypes of somatosensory neurons. This advancement has the potential to significantly enhance the effectiveness of symptom management or CIPN prevention while maintaining the efficacy of anti-cancer therapies. In this study, we aim to deepen the understanding of paclitaxel-induced neuropathic pain by employing single-nuclei RNA sequencing technology. This study may contribute to the development of mechanism-oriented approaches for treating CIPN.

## Materials and methods

### Animals and CIPN model

C57BL/6j mice weighing between 20 and 22 g were provided by the Guangdong Province Laboratory Animal Center (Guangzhou, China), maintained and bred in a normal 12 h light/dark cycle with standard feed and water. Experiments were performed using 8- to 12-week-old littermates. All experiments for snRNA-seq utilized male mice (n = 6 per group), while functional validation studies (Camk1d knockdown) included both male and female mice (n = 3 per sex). All experimental procedures were approved by the Animal Care and Use Committee of the 6th Affiliated Hospital of Shenzhen University Medical School in advance and were strictly in compliant with the guideline for the care and use of laboratory animals. Paclitaxel was dissolved in a solvent containing mix of DMSO-PEG300-Tween 80-Saline (2:4:1:13) according to the previous literature (Song et al. 2022). Each mouse received 4 mg/kg of paclitaxel every other day, for a cumulative intraperitoneal dosage of 32 mg/kg, or vehicle control.

## Behavioral analysis

### Von Frey test

Mechanical paw withdrawal threshold was assessed with eight von Frey hairs of different bending forces (0.008, 0.02, 0.04, 0.07, 0.16, 0.4, 0.6, and 1.0 mg) as we previously reported (Wu et al. 2023). A positive response consisted of flexion, leg raising, or foot licking behavior upon applying the filament to the hind paw. Mice were placed individually in a clear acrylic behavioral chamber. After habituated at least 20 min, von Frey hair was applied to the lateral plantar side of the right hind paw for 3–5 s to measure the mechanical threshold. Each filament was applied at most 5 times, switched to the next filament of either smaller force (if positive response occurred 3 times), or bigger force (if negative response occurred 3 times).

### Hot/cold-plate test

Thermal hyperalgesia and clod allodynia were assessed in C57BL/6 using a hot/cold plate analgesia meter (BIO-CHP, Bioseb, France) as we previously reported (Sun et al. 2018). The hot and cold plates were maintained at 53 ± 1 °C and 4 ± 1 °C, respectively. The time taken from the moment that exposing the mid-plantar surface of the hind paw on the hot/cold plate to the first nociceptive response including paw licking, lifting, or jumping was regarded as the thermal/cold withdrawal latency and recorded in seconds. A cut off time of 20 s was set to avoid tissue damage. A maximum stimulation time of 30 s was adopted to avoid tissue injury. Each mouse was measured 3 times with a 10-min interval.

### Nuclei isolation

Nuclei were isolated from mouse dorsal root ganglia (DRGs) as described previously with minor modification (Jung et al. 2023). DRGs were dissected in a 1.5 ml Eppendorf tube containing homogenization buffer (20 mM Tris–HCl pH 8.0, 500 mM sucrose, 50 mM KCl, 10 mM MgCl_2_, 0.1 mM DTT, 1% BSA, 0.1% NP40) supplemented with 1 × protease inhibitor cocktail (Roche, Indianapolis, USA) and 0.4 U/μl RNase inhibitor (Invitrogen, Carlsbad, USA). Tissues were transferred into a 2 ml Dounce Tissue Grinder (Kimble Chase, Rockwood, USA) and homogenized with A (“loose”) pestle for 20 strokes, followed with 20 strokes using B (“loose”) pestle on ice. The solution was filtered using a 70-µm cell strainer (Miltenyi Biotec, Maryland, USA) into a 15 ml tube, then filtered with a 30-µm cell strainer (Miltenyi Biotec, Maryland, USA). Nuclei were pelleted with centrifugation at 300 g for 5 min at 4 °C and resuspended in 1.5 ml PBS supplemented with 1% BSA and 0.2 U/μl RNase inhibitor (Invitrogen, Carlsbad, USA). The nuclear suspension was centrifuged at 300 g for 5 min at 4 °C and the pellet was resuspended with 1.5 ml cold PBS.

### Single-nucleus RNA-seq library preparation and sequencing

The nuclei were loaded into microfluidic chip of Chip A Single Cell Kit v2.0 (MobiDrop, Hangzhou, China) to generate droplets with MobiNova-100 (MobiDrop, Hangzhou, China). Each nuclear was wrapped into a droplet which contained reaction reagent and a gel bead linked with up to millions oligos (cell unique barcode). After encapsulation, droplets suffer light cut by MobiNovaSP-100 (MobiDrop, Hangzhou, China) following oligos diffusion into reaction mix. The mRNAs were captured by gel beads with cDNA amplification in droplets. Following reverse transcription, cDNAs with barcodes were amplified, and a library was constructed using the High Throughput Single Cell 3’RNA-Seq Kit v2.0 (MobiDrop, Hangzhou, China) and the 3'Single Index Kit (MobiDrop, Hangzhou, China). The snRNA libraries were sequenced on GenoLab M platform (GeneMind Biosciences, Shenzhen, China) via PE150 model (Pavel et al. 2023).

### The fluorescence in situ hybridization (FISH)

FISH assay was performed using a Fluorescent In Situ Hybridization Kit (Servicebio, Wuhan, China) following the manufacturer’s protocols. The hybridization utilized Camk1d (Camk1d probe sequence 5′- GGGTAACCACAGAGCAAGATATAGGCG-3′) with Alexa Cy3-labeled and 488-labeled probes (Obio Technology, Shanghai, China), respectively. Following prehybridization, the Camk1d probes were hybridized in the prepared hybridization buffer, and the nuclei were stained with DAPI (Yeasen, Shanghai, China). Fluorescence images were captured using a laser confocal microscope (Olympus, FV3000, Tokyo, Japan). All the images were obtained using the same acquisition settings, five DRG sections were randomly selected for data analysis. The intensity of the fluorescence was quantified using Image J software (NIH, Bethesda, MD, USA).

### AAV virus preparation

Camk1d silencing was achieved by subcloning rAAV-U6-shRNA (mCamk1d)-CMV-EGFP-WPREs from Brain Case (Shenzhen, China) into the AAV9 vector containing shRNA cassettes for Camk1d-shRNA, yielding a virus titer of 1.00E + 13 VG/mL. Additionally, a scrambled control rAAV-U6-shRNA (Scramble)-CMV-EGFP-WPREs from Brain Case (Shenzhen, China) was included in the experiment. shRNA (mCamk1d) primer sequences: Forward primer: caccgcagcctggacagttcaaatgttcaagagacatttgaactgtccaggctgc; Reverse primer: aaaagcagcctggacagttcaaatgtctcttgaacatttgaactgtccaggctgc.

### Intrathecal injection

Mice aged 6–7 weeks were subjected to intrathecal injection. After anesthesia with isoflurane (RWD, Shenzhen, China), the fur on the back of the mice was shaved from the tail to the caudal thoracic spine. Then, 7 μl of viral particles were injected into the L5-L6 intervertebral space using a 25 μl Hamilton syringe. Tail flicks were observed in mice as evidence of successful access to the dural cavity for intrathecal injection (Hylden and Wilcox 1980). Mice injected with rAAV-U6-shRNA or the scrambled control were allowed to recover for a minimum of 4 weeks before the modeling process.

### Statistical analysis

For behavioral tests, the data were analyzed in R and reported as Mean $\pm$ SEM. For gene expression, the results were presented in box and whisker plots. The middle line in the box represented the median of the data. The bottom and top of the box were the first (Q1) and third (Q3) quartiles, respectively. The whiskers extended from Q1 and Q3 to the most extreme data points, excluding outliers. Outliers were defined as data points less than or greater than 1.5*IQR (inter-quartile range) of Q1 or Q3 and displayed as black dots. Data were tested for assumptions of normality (Shapiro–wilk normality test) and equal variance (Bartlett test of homogeneity of variance) before comparisons of means. When the data were normally distributed and had equal variance between the groups, a parametric one-way or two-way repeated-measures ANOVA followed by multiple comparisons corrected by Tukey’s Honest Significant Difference (HSD) was applied, otherwise a non-parametric ANOVA (Kruskal–Wallis test with Conover-Iman test to correct multiple comparisons) was used. An adjusted *p* < 0.05 was considered a statistically significant difference.

## Results

### Repeated administration of paclitaxel resulted in pain hypersensitivity in mice

**Fig. 1:**
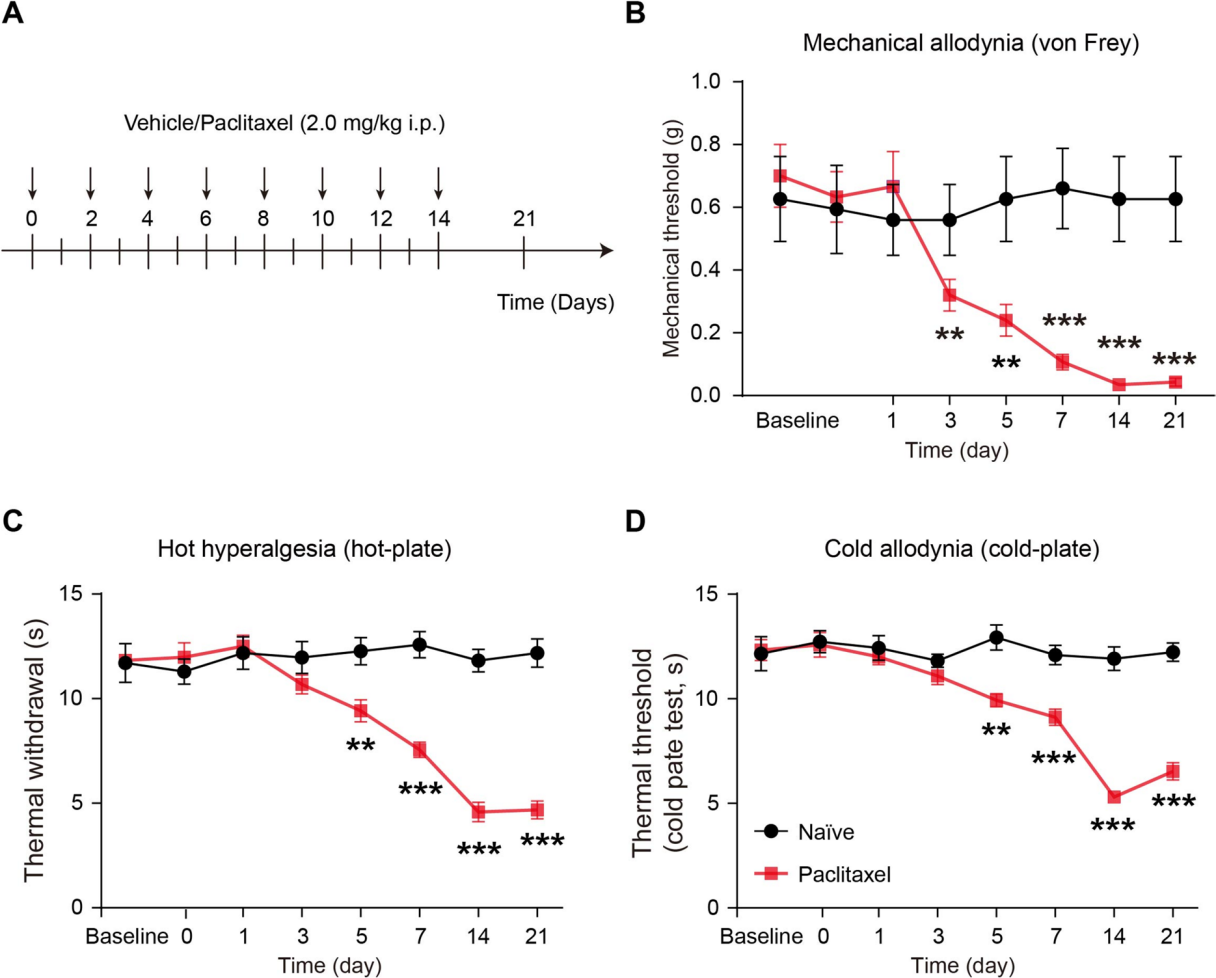
The development of a mouse model of paclitaxel-induced CIPN. A The process outline of development of a mouse model of paclitaxel-induced CIPN. B, C, and D Mechanical allodynia (B), thermal hyperalgesia (C) and cold allodynia (D) in mice following repeat administration of paclitaxel. The paw withdrawal thresholds in the von Frey test and response latencies in the hot and cold plate tests were measured before treatment (baseline) and after the first injection of either the vehicle or paclitaxel. The bar represents Mean ± SEM, with n = 6. The statistical significance is denoted as **p < 0.01, ***p < 0.001 vs. the naive group, and the statistical analysis was performed using two-way repeated-measures ANOVA with Tukey’s HSD correction

We established a mouse model of paclitaxel-induced neuropathic pain according to the outlined procedure in Fig. 1A. We assessed mechanical allodynia, as well as thermal hyperalgesia and cold allodynia post-paclitaxel administration. As expected, a significant decrease in mechanical threshold was observed on day 3 post-injection, reaching the lowest levels on day 14 post-injection (Fig. 1B). Meanwhile, paw withdrawal latencies in response to thermal or cold stimulation declined on day 5 post-injection and were most pronouncedly affected on day 14 post-injection (Fig. 1C and D). These observations indicate that the repetitive administration of paclitaxel induced allodynia and hyperalgesia in mice.

### The neuronal and non-neuronal composition of DRG

**Fig. 2:**
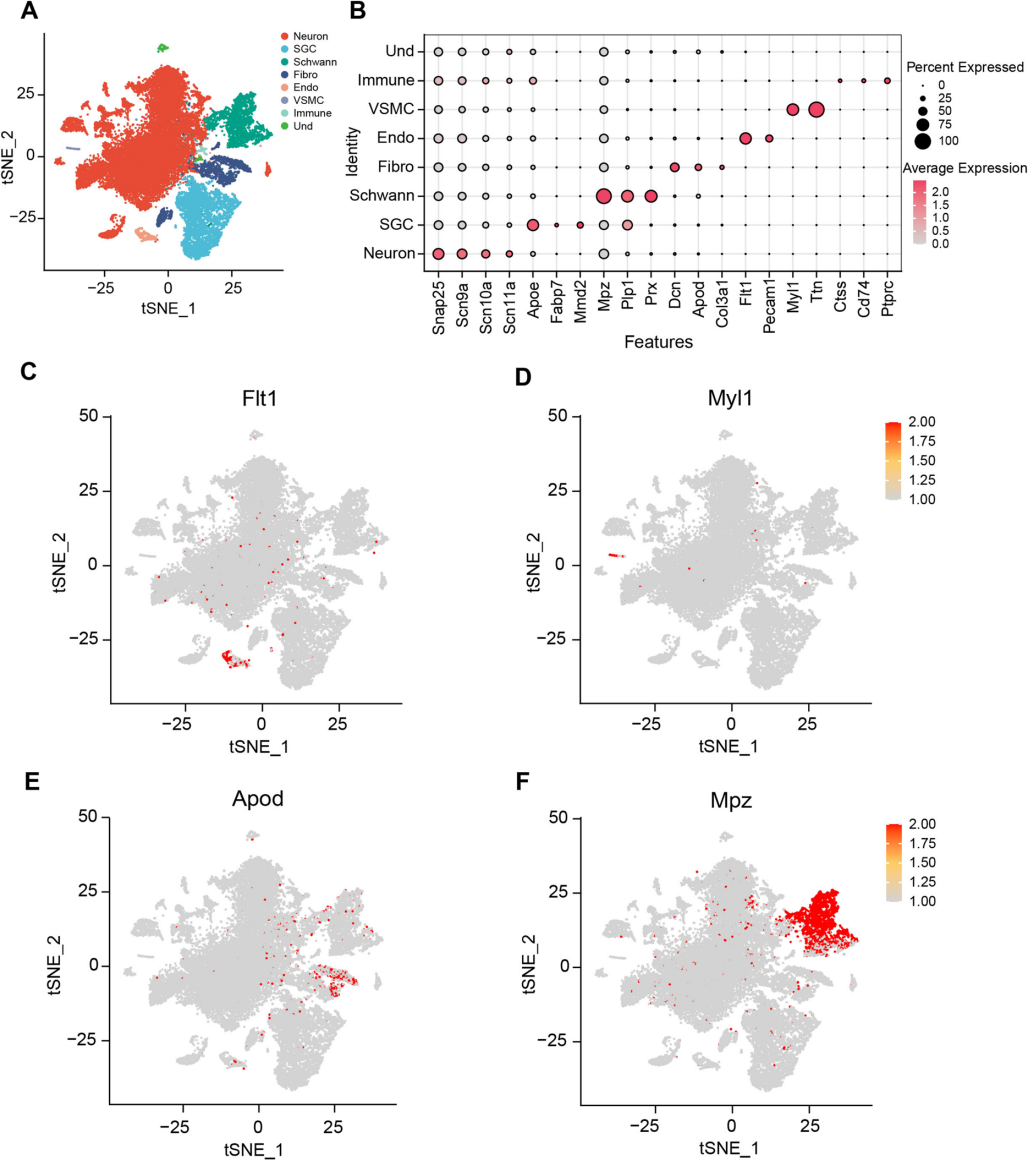
**The heterogeneity of dorsal root ganglia (DRG) cells in mice with paclitaxel-induced CIPN. A** t-distributed scholastic neighbor embedding (t-SNE) plot of single cells profiled in the study, with each cell type represented by a colored dot. **B** The dot plot of the cell types in the DRG of paclitaxel-induced CIPN mice, marked by distinct genes. **C, D, E, and F** t-SNE plots of single cell types of Endo (C), VSMC (D), Fibro (E), and Schwann cells (F), respectively

To investigate cell type-specific pathogenesis in mice with paclitaxel-induced neuropathic pain, we conducted a single-nuclei RNA sequencing on lumbar (L) 4, 5, and 6 DRGs. The DRGs were isolated from mice (n = x/group) subjected to treatments involving saline for 14 days (referred to Ctrl), paclitaxel treatment for 14 days (referred to Pac14) and paclitaxel treatment followed by a recovery period of 7 days (referred to Pac21). The Fastq files were pre-processed and then mapped with the mouse mm10 reference genome to create expression matrix, cells and genes documents using MobiVision V3 software (MobiDrop). The processed data were loaded separately to the Seurat package (V5) and merged without integration. We filtered out cells exhibited mitochondrial genes percentage exceeding 5%, RNA counts fewer than 500, RNA counts over fivefold of the median RNA counts, and number of features exceeding fivefold of the median RNA features. The merged data underwent analysis using “LogNormalize” normalization and “pca” dimension reduction. Subsequently, the first 30 dimensions were input for constructing SNN group, with a resolution of 0.1 applied to differentiate various cell clusters. A total of 8 major cell populations were identified in the DRG (Fig. 2A), including Snap25 + neurons, Apoe + satellite glial cells (SGC), Mpz + Schwann cells (Schwann), Dcn + Fibroblasts (Fibro), Flt1 + endothelia cells (Endo), Myl1 + vascular smooth muscle cells (VSMC), and Ptprc + immune cells (Immune) (Fig. 2B-2F). One cluster could not be defined and categorized as undetermined (Und, possibly contaminated cells) due to a lack of known cell type-specific markers (Fig. 2B).

### The somatosensory neuron clusters in DRG

**Fig. 3:**
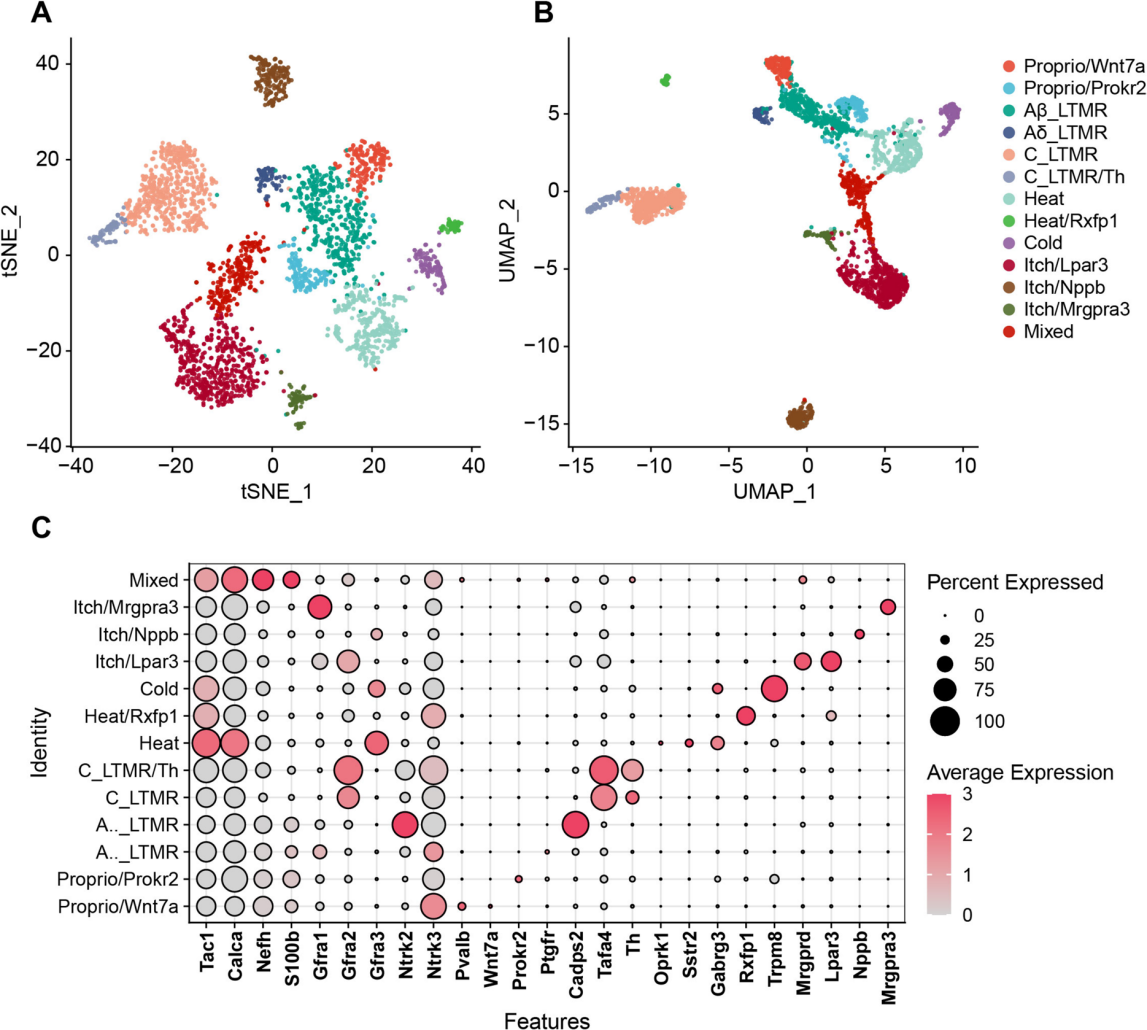
**The heterogeneity of DRG neurons in mice with paclitaxel-induced CIPN. A and B** t-SNE (A) and UMAP (B) plots of single cells profiled in the study, with each neuron type represented by a colored dot. **C** The dot plot of the neuron types in the DRG of paclitaxel-induced CIPN mice, marked by distinct genes

We then focused on the neuron cluster and utilized the list of DRG neuron markers summarized by Wang et al. (Wang et al. 2021b) to discern distinct neuronal populations within the DRG. We subset the counts of neuron cluster and perform a similar analysis as described above. The first 30 dimensions were used in PCA reduction and a resolution of 0.5 was set to distinguish neuronal subclusters. We noticed that two clusters (cluster 0 and 1) had very low RNA counts (counts of all cells < 1000) and lacked specific markers. These cells were considered low quality and removed by setting the threshold at nCount_RNA > 1000. The remaining data were then re-analyzed. We successfully identified 12 neuronal clusters, including Wnt7a + proprioceptors (Proprio/Wnt7a), Prokr2 + proprioceptors (Proprio/Prokr2), Ptgfr + Aβ low-threshold mechanoreceptors (Aβ_LTMR), Cadps2 + Aẟ_LTMR, Tafa4 + C_LTMR, Th +/Tafa4 + C_LTMR/Th, Sstr2 + thermal nociceptors (Heat), Rxfp1 + thermal nociceptors (Heat/Rxfp1), Trpm8 + cold nociceptors (Cold), Mrgprd +/Lpar3 + pruriceptors (Itch/Lpar3), Nppb + pruriceptors (Itch/Nppb) and Mrgpra3 + pruriceptors (Itch/Mrgpra3). A small cluster included multiple markers and was considered a mixed cell cluster. The t-SNE and UMAP coordinates of all neurons were plotted and demonstrated (Fig. 3A-3B). The dot plot of neuronal subtype-specific markers was also displayed (Fig. 3C).

### The gene ontology (GO) annotation of DEGs suggested potential alterations in nerve fiber and potassium-related currents in C_LTMR following paclitaxel treatment

**Fig. 4:**
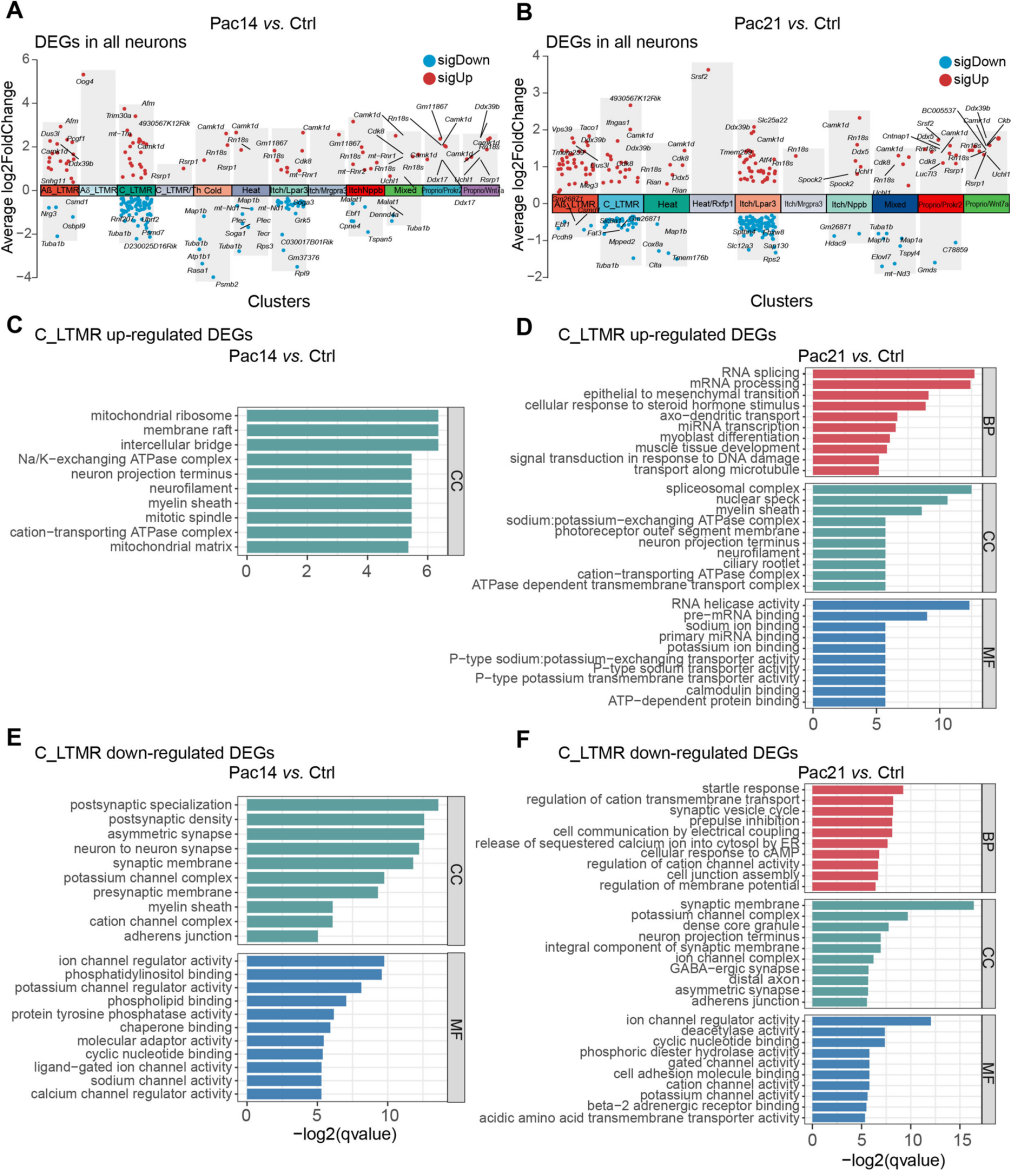
**The transcripts regulated in all DRG neurons in mice with paclitaxel-induced CIPN. A and B** The volcano plots showing the up- and down-regulated DEGs (differentially expressed genes) in all types of neurons in the DRG at both 14 (A) and 21 (B) days after paclitaxel injection. **C and D** The comparison of gene ontology (GO) enrichment of the up-regulated DEGs in C_LTMR neurons following paclitaxel injection at 14 (C) and 21 (D) days in mice. **E and F** The comparison of gene ontology (GO) enrichment of the down-regulated DEGs in C_LTMR neurons following paclitaxel injection at 14 (E) and 21 (F) days in mice. Top 10 significantly enriched GO terms, including biological process, cellular component, and molecular function, are shown

We then performed the analysis of differentially expressed genes (DEGs) across all neuronal subclusters between Ctrl and Pac14 or Pac21. The gene expression in the minimum percentage of cells (min.pct) and the average log2 fold change (avg_logFC) between groups were set at 0.25. The results unveiled notable up- and down-regulated DEGs across all neuron types within the DRG at both day 14 and 21 post-paclitaxel injection (Fig. 4A and B). Several genes, including Calcium/calmodulin dependent protein kinase 1D (Camk1d), 18S ribosomal RNA (Rn18s), and Cyclin dependent kinase 8 (Cdk8) were commonly up-regulated in multiple neuronal subtypes at two time points, indicating that these genes may be markers of paclitaxel exposure. Surprisingly, at the current sequencing depth, we were unable to quantified the expression of Atf3 gene (counts of all cell = 0), a marker of neuronal injury. It is worth noting that the C_LTMR and Itch/Lpar3 cluster had more DEGs compared to other clusters at both time points (Fig. 4A and B), suggesting that these two clusters were most vulnerable to paclitaxel treatment at the transcriptome levels. In this study, subsequent analysis focused on C_LTMR, which has been associated with pain hypersensitivity in many models of chronic pain.

Gene ontology (GO) analysis of the DEGs in the C_LTMR cluster was then performed. Analysis of up-regulated DEGs at day 14 post-paclitaxel injection failed to enrich biological processes (BP) and molecular function (MF) terms, but highlighted the association of cellular component (CC) terms such as"mitochondrial ribosome","mitochondrial matrix", and"neuron projection terminus"(Fig. 4C), suggesting that mitochondrion and axonal fibers of C_LTMR might have been affected. At day 21 post-injection (Fig. 4D), enriched biological processes encompassed"RNA splicing","mRNA processing", and"signal transduction in response to DNA damage"in C_LTMR neurons. Enriched cellular component terms included"spliceosomal complex","neuron projection terminus", and"neurofilament", while enriched molecular functions were linked to"RNA helicase activity","pre-mRNA binding"and"calmodulin binding". These results indicated that there may be persistent impacts on axonal fibers of C_LTMR and reorganization of RNA processing during paclitaxel recovery.

For down-regulated DEGs, enriched cellular component terms such as"postsynaptic specialization","postsynaptic density","neuron to neuron synapse", and"potassium channel complex"were revealed at 14 days post-injection (Fig. 4E). At 21 days post-injection (Fig. 4F), enriched biological processes encompassed"synaptic vesicle cycle","cellular response to cAMP", and"regulation of membrane potential"in C_LTMR neurons. Additionally, enriched cellular component terms included"synaptic membrane","potassium channel complex","GABAergic synapse", and"asymmetric synapse", while enriched molecular functions were associated with"ion channel regulator activity","deacetylase activity", and"potassium channel activity". This analysis implies that paclitaxel treatment could lead to dysregulation in potassium related currents, which might subsequently affect the excitability of C_LTMR.

### Gene set enrichment analysis (GSEA) of transcriptomic changes in C_LTMR neurons

**Fig. 5:**
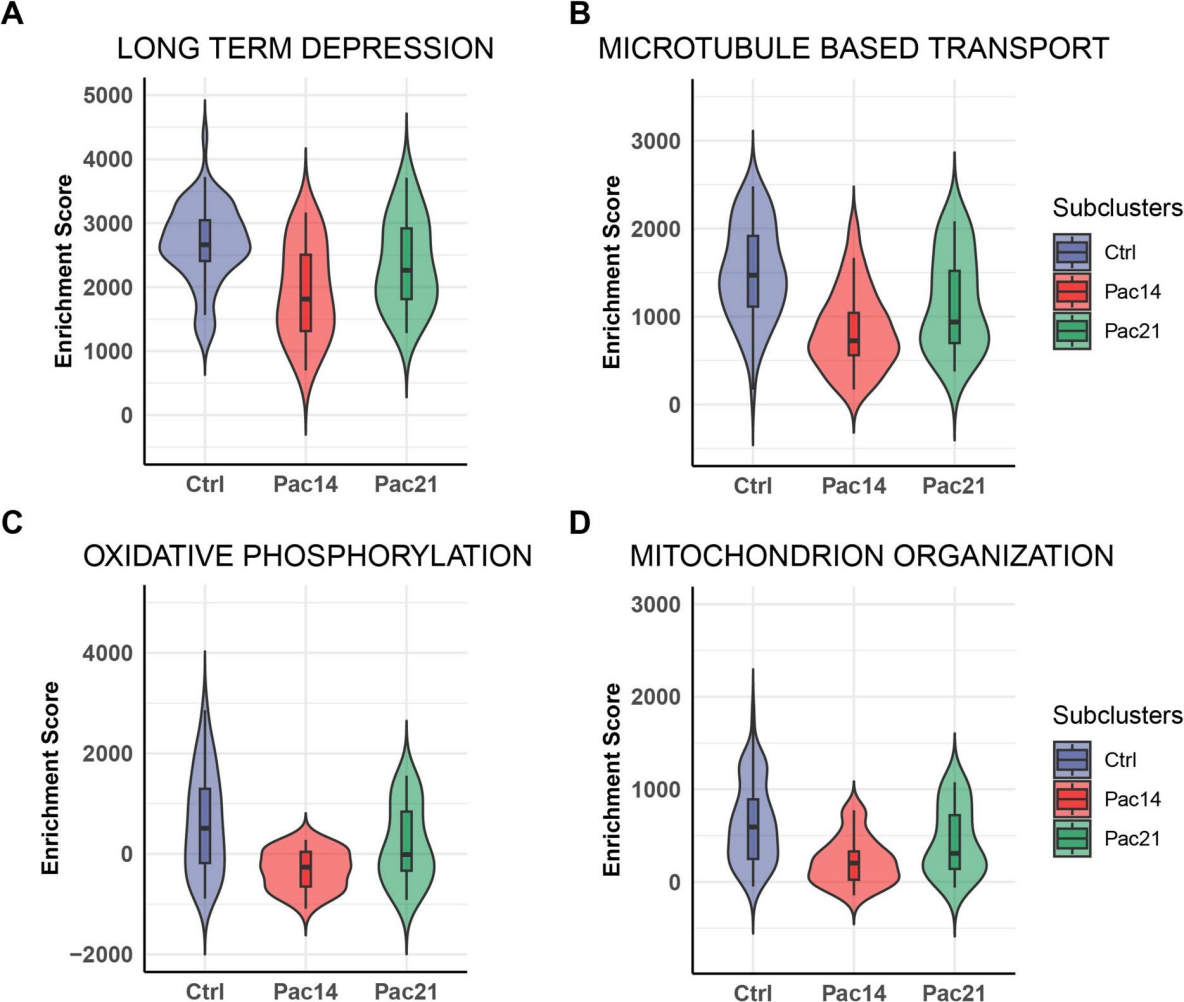
**The gene set enrichment analysis of transcriptomic changes in C_LTMR neurons. A, B, C, and D** The desregulated terms in C_LTMR neurons in mice, showing that paclitaxel treatment was associated with several biological processes, such as"long-term depression"(A),"microtubule-based transport"(B),"oxidative phosphorylation"(C), and"mitochondrion organization"(D). **E** The enrichment score of Interleukin 17 production in C_LTMR neurons in mice after paclitaxel treatment (E)

While enrichment analysis using DEGs reveals prominent treatment-related ontologies, it may overlook pathways associated with DEGs exhibiting subthreshold fold changes. To address this limitation, we employed the GSEA method to identify affected Gene Ontology Biological Processes (GOBPs) in C_LTMR cluster. The Seurat object of neurons was loaded to escape (Easy single cell analysis platform for enrichment) package and subjected to GOBP enrichment analysis. As depicted in Fig. 5, aligning with the well-defined effects of paclitaxel treatment, the enrichment scores of several biological processes, including “microtubule-based transport”, and “oxidative phosphorylation,” and “mitochondrion organization” were significantly decreased in C_LTMR neurons treated with paclitaxel for 14 days compared to saline (Fig. 5A, B, C, and D). This result confirms the disruption of microtubule-related functions and reveals downstream mitochondrial deficits in C_LTMRs in CIPN mouse, providing a pathway-centric explanation for neurotoxicity.

### Pseudo-time analysis of C_LTMR cluster

**Fig. 6:**
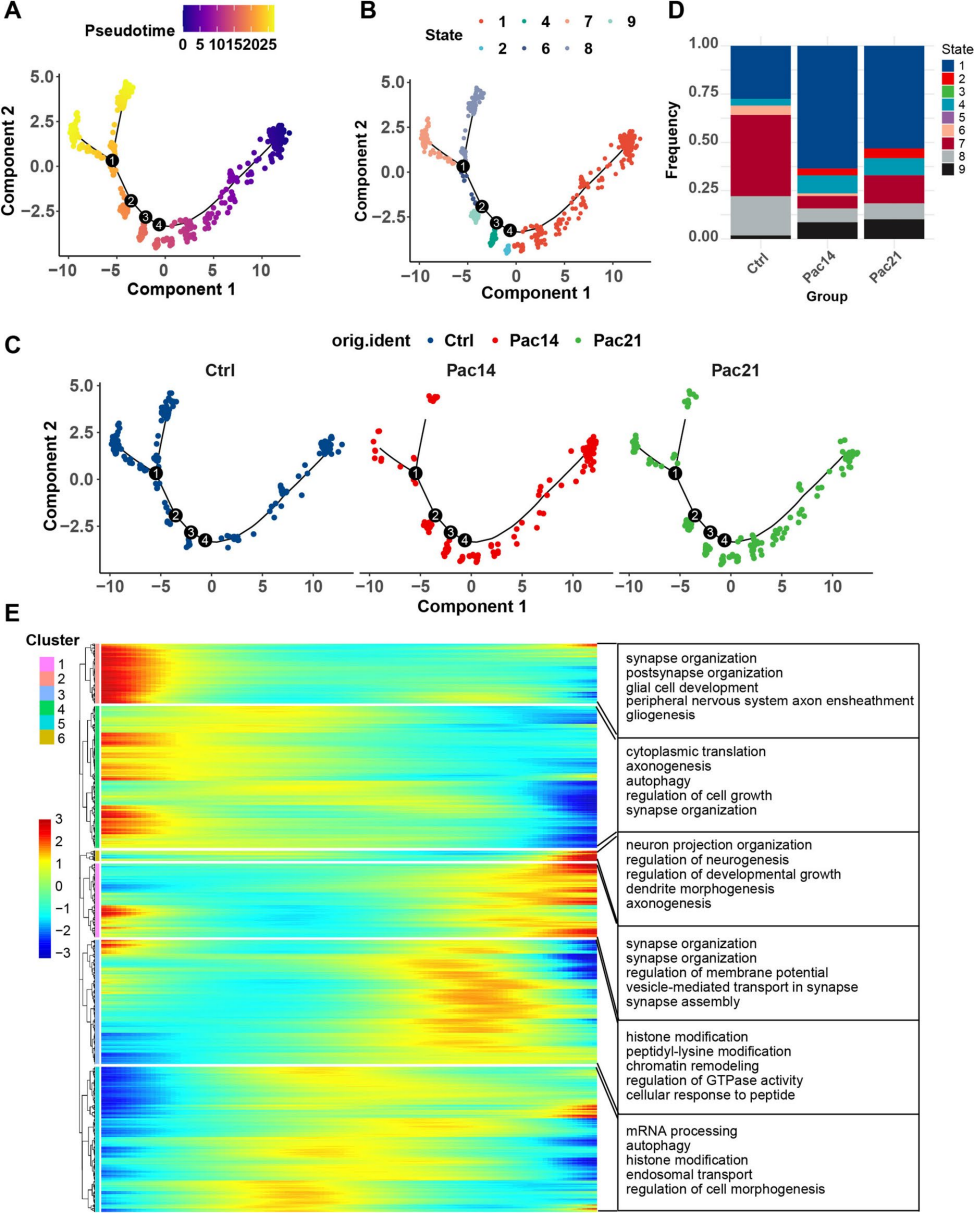
**Pseudotime analysis of transcriptomic changes in C_LTMR neurons. A** Pseudo-time trajectories show the transition of the process in paclitaxel-induced CIPN mouse DRG C_LTMR neurons. The dots represent cells, and the colors indicate neuron clusters or pseudotime. **B** Genes in C_LTMR neuronal switch process are classified into 9 modules based on their expressing patterns. **C** The separation of Modules 1, 2, and 3. **D** The bar graph showing the frequency ratio of each module. **E** Heatmaps that display the DEGs module based on their dynamic expression characteristics, as shown in pseudotime analysis, in C_LTMR neurons in paclitaxel-induced CIPN mice. Gene ontology (GO) enrichment analysis results were used to analyze the biological processes of each gene module

Pseudo-time analysis enables the discovery of different cell states that exhibit subtle differences in cellular behaviours within a cell population. We subset the C_LTMR cluster and constructed pseudo-time trajectories to identify different cell states after paclitaxel treatment and genes with altered expression as the neurons transitioned between states (Fig. 6A). A total of 9 states were identified across the trajectories (Fig. 6B and C). Interestingly, the distribution of cell states varies among different groups, notably with a substantial increase in the prevalence of state 1 observed in two paclitaxel treatment groups. This result suggest that C_LTMR in state 1, positioned at the earliest point of the pseudo-time trajectory, represent a responsive state following paclitaxel exposure (Fig. 6B-D). We then performed unsupervised clustering analysis of gene expression and categorized genes according to their expression trends across the pseudo-time frame. This assigned genes into 6 different clusters. Notably, genes in cluster 2 and 4 exhibited a decreasing trend, whereas cluster 1 demonstrated an increasing pattern across the pseudo-time trajectory, indicating a close association with state 1 (Fig. 6E). The GO annotations of clustered genes show enrichment for various neurotransmission related terms, such as “synapse organization”, “axonogenesis”, “regulation of membrane potential” and “vesicle-mediated transport in synapse”. On the other hand, genes in clusters less likely associated with state 1 were more relevant to transcription modulation (e.g. “histone modification” and “chromatin “remodelling”). This result implies that the sustained presence of state 1 could directly contribute to abnormal pain processing after paclitaxel administration (Fig. 6E).

### Gene interaction analysis unveiled gene modules associated with altered biological processes in state 1 C_LTMR

**Fig. 7:**
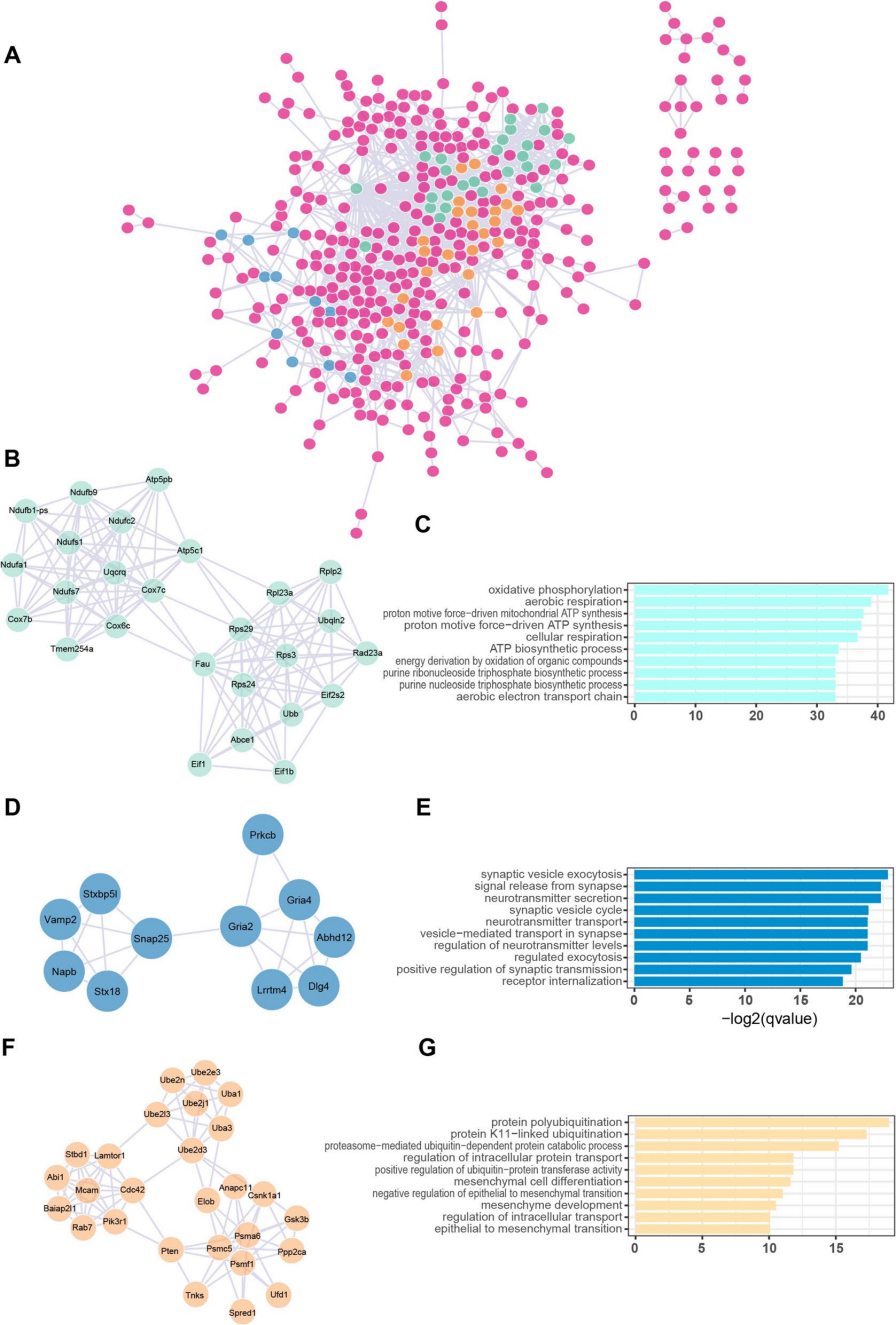
**Gene interaction analysis in state 1 C_LTMR neurons in mice with paclitaxel-induced CIPN. A** Protein–protein interaction (PPI) network generated from genes in cluster 2, corresponding to pseudotime state 1. The network was constructed using STRING and analyzed with the MODE algorithm to identify functional modules. Nodes represent genes, and subnetworks identified as significant by MODE are shown in distinct colors, while non-significant subnetworks are displayed as purple nodes. **B, D, and F** The MCODE plugin of Cytoscape software revealed numerous densely connected subnetworks, notably highlighting subnetwork 1 (B), subnetwork 2 (D), and subnetwork 3 (F). **C, E, and G** GO analyses were conducted to determine the biological processes associated with each of these subnetworks 1 (C), subnetwork 2 (E), and subnetwork 3 (G) in state 1 C_LTMR neurons in mice with paclitaxel-induced CIPN

The products of genes interact physically or co-occur to accomplish biological processes. To identify gene modules potentially involved in altered biological processes in state 1 C_LTMR, we extracted the genes from cluster 2 (Fig. 6E), which might have relevance to state 1, and constructed a gene interaction network using STRING (Fig. 7A). The MCODE plugin of Cytoscape software revealed numerous densely connected subnetworks, notably highlighting subnetwork 1, which could explain changes related to mitochondrion (Fig. 7B and C), and subnetwork 2, which could be relevant to alterations in neurotransmission (Fig. 7D and E). Additionally, the existence of subnetwork 3 also suggest that paclitaxel might dysregulate the ubiquitination system in C_LTMR cluster (Fig. 7F and G).

### The up-regulation of camk1d contributed to thermal hyperalgesia and cold allodynia in mice with paclitaxel-induced CIPN

**Fig. 8:**
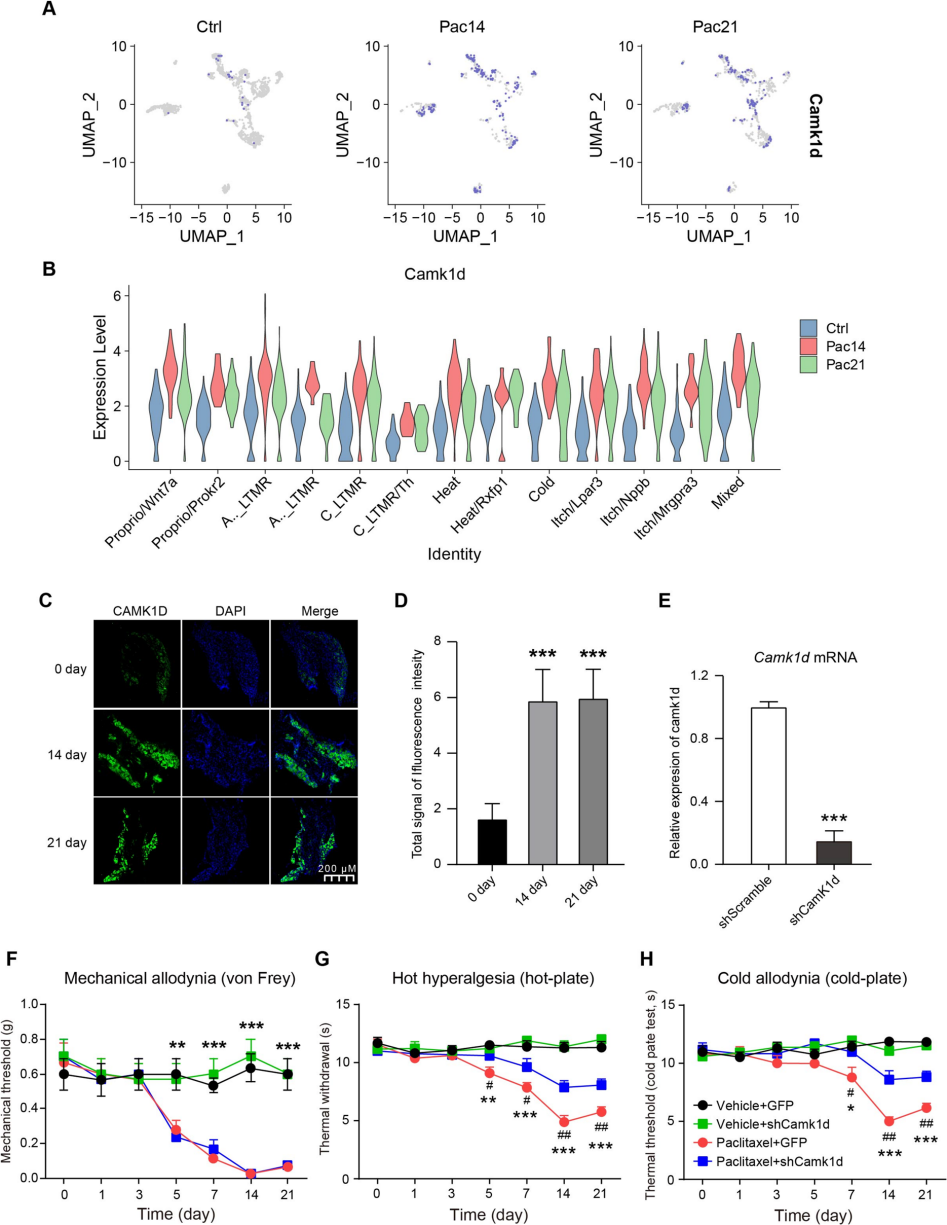
**The expression and function of Camk1d in DRG in mice with paclitaxel-induced CIPN. A** Feature plots of Camk1d expression using UMAP for dimensionality reduction. **B** Violin plots illustrating its upregulation following paclitaxel treatment. **C** In situ hybridization images exhibit the expression of Camk1d (green) and DAPI (blue) in DRG in mice with 0, 14, and 21-day paclitaxel-induced CIPN. **D** The quantification of the fluorescence signal from panel A. **E** The mRNA expression of *Camk1d* in DRG in mice after Camk1d knockdown. **F, G, and H** Mechanical allodynia (F), thermal hyperalgesia (G) and cold allodynia (H) in mice with or without knockdown of Camk1d following repeated administration of paclitaxel. The paw withdrawal thresholds in the von Frey test and response latencies in the hot and cold plate tests were measured before treatment (baseline) and after the first injection of either the vehicle or paclitaxel. Mean ± SEM, n = 6. The statistical significance is denoted as **p < 0.01, ***p < 0.001 *vs.* the GFP + saline group, ^#^*p* < 0.05, ^##^*p* < 0.01 *vs.* the PTX + Camk1d group and the statistical analysis was performed using two-way repeated-measures ANOVA with Tukey’s HSD correction

Camk1d was commonly upregulated across multiple neuronal subtypes at two time points, suggesting its potential role as a biomarker of paclitaxel exposure. Feature plots (using UMAP dimensionality reduction) and violin plots illustrated this upregulation in paclitaxel-treated DRG neurons (Fig. 8A and B). This increase was confirmed by in situ hybridization in the DRG of mice with paclitaxel-induced CIPN, which demonstrated upregulation in multiple neuronal subtypes at both time points (Fig. 8C and D). To further investigate the function of Camk1d in CIPN, we performed Camk1d knockdown in the DRG of CIPN mice (Fig. 8E). The results reveal that mechanical allodynia (increased sensitivity to touch) was unaffected by Camk1d knockdown (Fig. 8F). However, Camk1d knockdown significantly reduced thermal hyperalgesia (increased sensitivity to heat) and cold allodynia (increased sensitivity to cold) in response to thermal or cold stimuli (Fig. 8G and H). These findings indicate that Camk1d contributes to temperature hypersensitivity, a key clinical symptom of CIPN in humans. In contrast, Camk1d does not appear to mediate mechanical allodynia in this mouse model of CIPN.

## Discussion

### C_LTMRs as key mediators of paclitaxel neurotoxicity

CIPN remains a debilitating complication of cancer treatment, often necessitating dose reduction or discontinuation of therapy, thereby undermining patient outcomes. Paclitaxel, a cornerstone chemotherapeutic agent, induces CIPN in up to 100% of patients, with symptoms persisting for months or even years post-treatment (Saif and Reardon 2005). This study employs snRNA-seq to elucidate the molecular mechanisms underlying paclitaxel-induced CIPN, revealing C_LTMRs as key mediators in its pathogenesis. Our findings highlight transcriptomic disruptions affecting potassium currents, microtubule dynamics, and mitochondrial function within C_LTMRs, alongside novel roles for Camk1d in thermal hypersensitivity. These insights not only enhance our mechanistic understanding but also identify neuron-specific therapeutic targets, addressing a critical gap in CIPN management.

### Pathways linking transcriptomics to function

GO and GSEA collectively delineate three interconnected pathological pathways activated in C_LTMRs following paclitaxel treatment: (1) suppression of potassium channel activity, evidenced by the downregulation of Kcnq and Kcna families (Fig. 4E-F), which likely elevates neuronal excitability, as demonstrated in bortezomib-induced neuropathy (Verma et al. 2020; Wu et al. 2022); (2) microtubule transport impairment, where paclitaxel’s primary mechanism of microtubule stabilization paradoxically disrupts axonal trafficking (Fig. 5B), thereby exacerbating mitochondrial dysfunction (Staff et al. 2013); and (3) mitochondrial stress, characterized by impaired oxidative phosphorylation (Fig. 5C-D), which aligns with the metabolic crisis observed in CIPN (Canta et al. 2015; Zhai et al. 2023). These pathways are mechanistically intertwined—microtubule destabilization may hinder mitochondrial trafficking, amplifying oxidative stress and energy deficits. Notably, at both day 14 and day 21 after paclitaxel treatment, there was enrichment of terms related to synaptic function and neurotransmission. Concurrently, downregulated genes were significantly enriched for potassium channel-related terms (e.g., “potassium channel complex” and “potassium channel activity”). Dysregulation of potassium channels is a well-established mechanism contributing to neuronal hyperexcitability. Reduced expression or function of key Kv subunits, such as Kv1.2 (Kcna2) and Kv7.2 (Kcnq2), can impair membrane repolarization and lower the threshold for neuronal firing and increasing excitability, resulting in increased spontaneous activity and heightened pain sensitivity (Wang et al. 2021a; Yee et al. 2022). These alterations in ion channel expression and neuronal signaling likely underlie the hyperexcitability observed in chemotherapy-induced peripheral neuropathy.

### Heterogeneity and roles of other neuron subtypes

In addition to the nociceptive “C-LTMR” clusters emphasized in this study, the “Aβ-LTMR” subtype also exhibited a substantial number of DEGs at both 14 and 21 days post-paclitaxel administration (Fig. 5A and B), although we did not explore this population in depth. Previous studies have shown that both myelinated (A-fibers) and unmyelinated (C-fibers) responses to peripheral mechanical stimuli in CIPN animal models (Zhang and Dougherty 2014). The pronounced hyperexcitability of Aβ and Aδ neurons in the DRG has been implicated in key sensory abnormalities in paclitaxel-treated models, including tactile allodynia (Xiao and Bennett 2008). Further investigations are required to understand the roles of Aβ subtypes as potential contributors to tactile hypersensitivity in CIPN. Notably, within C_LTMRs, the TH-positive (TH +) and TH-negative (TH −) subpopulations exhibited divergent responses: the TH − neurons demonstrated significant mitochondrial dysfunction (Fig. 4C-F). These differences reflect heterogeneity documented in previous rodent models (Wang et al. 2023), suggesting that TH − neurons may underpin maladaptive neuroplasticity—shifting from affective touch to nociceptive signaling—thus contributing to the persistent pain phenotypes observed clinically, where innocuous stimuli are perceived as painful.

### Camk1d as a thermal-specific therapeutic target

Amk1d emerged as a pivotal mediator of thermal hypersensitivity, with its knockdown selectively reversing heat and cold hyperalgesia (Figs. 8G-H) but sparing mechanical hypersensitivity. Its upregulation in C_LTMRs, along with its expression in Heat/Rxfp1 and Cold neuron subtypes (Fig. 8A–B), suggests a calcium-dependent mechanism. CamK1d, as a calcium/calmodulin-dependent protein kinase, may sensitize TRPV1 and TRPM8 channels or enhance voltage-gated calcium channel (VGCC) activity, thereby augmenting nociceptor excitability (Zamponi et al. 2015). The modality-specific response correlates with the minimal expression of Camk1d in Aβ/Aδ neurons and their resistance to transcriptional changes following paclitaxel. Future investigations should clarify whether Camk1d modulates calcium influx directly or through downstream effectors, such as CREB, and whether its inhibition can normalize hyperexcitability in CIPN. Our data indicate that CamK1d is expressed across multiple neuron subtypes. C-LTMRs are typically associated with the detection of gentle and affective touch as well as mechanical itch, the observed CamK1d-related thermal hypersensitivity is more likely be mediated through its activity in Aβ fibers. Further investigation is warranted to clarify the specific neuronal subtypes in which CamK1d contributes to CIPN.

### Limitations

A limitation of this study is the insufficient sex-stratified analysis, due to the small sample size in the functional validation experiments (n = 3 per sex), which restricts our ability to detect potential sex-dependent effects in paclitaxel-induced neuropathy. Existing preclinical and clinical studies have produced conflicting findings—some report greater cold allodynia in female mice (Miguel et al. 2022; Naji-Esfahani et al. 2016), while others find no significant sex differences in clinical cancer pain (Ahmed et al. 2017). Sex is therefore an important clinical variable that warrants further investigation to inform pain management strategies following chemotherapy. We plan to address this in future studies using larger, sex-balanced cohorts to clarify these discrepancies. Furthermore, the snRNA-seq cohort was conducted exclusively in males, preventing assessment of sex differences. This is particularly relevant given the established sexual dimorphism in CIPN prevalence and severity (Hershman et al. 2014; Loprinzi et al. 2020).

While snRNA-seq provided unprecedented resolution of DRG neuron subtypes, several limitations remain. First, pseudotime analysis revealed a dominant “State 1” C_LTMR population following paclitaxel treatment (Fig. 6B-D); however, functional validation of these cellular states is still pending. Second, the intrathecal delivery of AAV9 (Fig. 8E) may have led to the silencing of Camk1d in spinal neurons, which could confound the interpretation of the results. Utilizing DRG-restricted knockdown models, such as AAV6 or AAV8, could help clarify the site-specific roles of Camk1d. Addressing these gaps is essential to enhance the translational relevance of the findings.

### Future therapies for CIPN

Our findings nominate Camk1d as promising targets for temperature-specific CIPN intervention. Inhibitors of Camk1d, especially when combined with potassium channel openers (e.g., retigabine) or microtubule stabilizers (e.g., epothilones), could potentially mitigate neurotoxicity without impairing the antitumor efficacy of paclitaxel. Furthermore, the heterogeneity of C_LTMR subpopulations advocates for subtype-selective therapeutic strategies. For instance, interventions targeting TH − neurons could alleviate neuropathic pain specifically, while leaving TH + -mediated affective touch unaffected.

## Concluding remarks

By integrating snRNA-seq data with behavioral and molecular validation, this study positions C_LTMRs as central orchestrators of paclitaxel-induced CIPN. The identified mechanisms, including suppression of potassium currents, microtubule dysfunction, and mitochondrial stress, coupled with Camk1d-driven thermal hypersensitivity, outline a multifaceted mechanistic landscape. These insights not only deepen our understanding of CIPN pathogenesis but also provide a roadmap for developing precision therapies targeting specific neuronal subtypes and pathways. Future research must aim to bridge the remaining gaps in functional validation, investigate sex-specific mechanisms, and elucidate spinal contributions, thereby ensuring that these discoveries are effectively translated into safer and more effective treatments for cancer survivors.

## Data Availability

The single-cell sequencing data from PIPN mice were available in the figshare database (27,627,786). DOI: 10.6084/m9.figshare.27627786
